# Neurological expression of an inherited translocation of chromosomal 1 and 7

**DOI:** 10.17712/nsj.2017.1.20160436

**Published:** 2017-01

**Authors:** Nabil A. AlMajhad, Amal M. AlHashem, Inesse A. Bouhjar, Brahim M. Tabarki

**Affiliations:** *From the Division of Pediatric Neurology (AlMajhad, Tabarki), Division of Genetics (AlHashem), Department of Pediatrics, Cytogenetics and Molecular Cytogenetics Laboratory (Bouhjar), Pathology and Laboratory Medicine, Prince Sultan Military Medical City, Riyadh, Kingdom of Saudi Arabia*

## Abstract

An unbalanced translocation of chromosome 1 and 7 (t[1;7]) associated with neurological phenotype and brain malformation has rarely been reported. This clinical report describes 3 siblings with brain malformations and a 13.5 Mb duplication of 1q42.3q44, and a 7.6 Mb duplication of 7q36.1q36.3 detected by array comparative genomic hybridization. This unbalanced t(1;7) was found to be inherited from a balanced translocation from the mother. All the patients presented with hypotonia, microcephaly, developmental delay, seizures, abnormal corpus callosum and abnormal cerebellum.

Unbalanced translocations involving chromosome 1 and 7 (t[1;7]) are well-recognized cytogenetic abnormalities in hematological malignancies and are often associated with poor prognosis.^1^ A literature search revealed that only a few cases of t(1;7) have been reported to be associated with neuropsychiatric manifestations or congenital brain malformation.^2^-^7^

Here we report 3 affected siblings with severe neurological phenotype and brain malformation with an unbalance t(1;7) inherited from a balanced translocation from the mother. Our objective in presenting this particular case is to compare our patients’ clinical and neuroimaging features with those of other cases reported in the literature. Our data can then contribute facilitating genotype-phenotype correlations.

## Case Report

**Figure 1:**
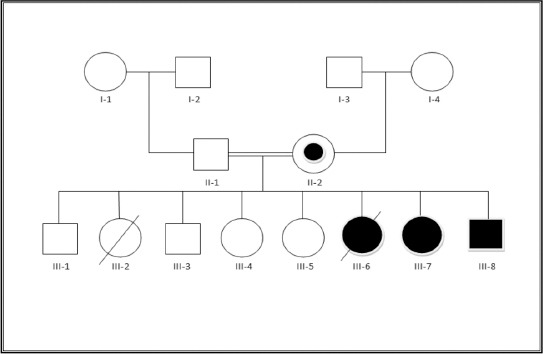
- The pedigree. The proband’s mother (II-2) was the balanced translocations carrier without clinical presentations. Patients III-1, III-3 and III-4 are normal. Patient III-2 had developmental delay, corpus callosum agenesis and congenital heart disease, but no genetic test was carried out for her. Patient III-6 died at the age of 6 years post cardiac surgery

We evaluated 3 patients from a consanguineous Saudi family (**Figure 1**). The main clinical, MRI, and associated malformations are summarized in **Table 1**.

**Table 1:** Clinical features of patients with translocation (1;7) and neurological phenotype.

Studies	Cytogenetic abnormality	Head/neck	Neurological features	Neuroradiology	Other malformations
Chuang et al^2^	t(1;7)(q32;q32)	Small mouth, hypotelorism, flat nose	Fetal period, termination of pregnancy	Alobar holoprosencephaly	Absence of ethmoidal and nasal bones
Schinzel^6^,^7^3 cases	t(1;7)(q32;q34)	Cyclopic features	NA	Holoprosencephaly	Hydronephrosis
Yan et al^7^	t(1;7)(p22;q21)	High Arched palate Narrowed mandible	Autism Schizophrenia	NA	NA
McPherson et al^4^et alet	t(1;7) (p22;q22)	High Arched palate Narrowed mandible	Autism Schizophrenia	NA	NA
McPherson et al^4^et alet	t(1;7)(q21.3;q34)	Coarse facial appearance, hirsutism, wide mouth, micrognathia	GDD, hypotonia, microcephaly	NA	Absent nails, hypoplastic 5^th^ finger
*Current study*					
Patient 1	t(1;7)1q42.3q44, 7q36.1q36.3	Wide forehead, hypotelorism	Seizures, GDD, microcephaly hypotonia	Agenesis of the corpus callosum, hypoplasia of the inferior vermis	Hypospadias Undescended testis
Patient 2	t(1;7)1q42.3q44, 7q36.1q36.3	Wide forehead, hypotelorism	GDD, microcephaly	Thick and abnormal corpus callosum, hypoplasia of the inferior vermis	Right club foot
Patient 3	t(1;7) 1q42.3q44, 7q36.1q36.3	Wide forehead, hypotelorism	Seizures, GDD, microcephaly	Atretic cephalocele, agenesis of corpus callosum, hypoplasia of the inferior vermis	NA

NA - not available, GDD - global developmental delay

Array-comparative genomic hybridization performed in patients III-6, III-7, and III-8 detected a deletion at 1q42.3q44 spanning 13.5Mb, and a duplication at 7q36.1q36.3 spanning 7.6Mb. The fluoresence in situ hybridization studies confirmed both the deletion and the duplication (1q42.3q44, 7q36.1q36.3). Studies of the parents showed normal karyotype in the father (46, XY), and an unbalanced reciprocal translocation in the mother 46,XX,t(1;7)(q44;q36). Whole exome sequencing to rule out other possible genetic causes was negative. Clinically, our patients had severe intrauterine growth restriction and oligohydramnios. They showed dysmorphism in the form of wide forehead, hypotelorism, upturned nose, and narrow anterior fontanelle. The neurological evaluation showed microcephaly (head circumference between -3 and -4 standard deviation) in all 3 patients. All patients had axial hypotonia and normal deep tendon reflexes. They showed global developmental delay with slow improvement. Patients III-6 and III-8 developed generalized tonic-clonic seizures at the age of 8 months, and the electroencephalogram showed multifocal discharges. Other non-neurological abnormalities included the following malformations: undescended testis, hypospadias, and congenital heart disease. The family had a history of 4 spontaneous abortions.

**Figure 2:**
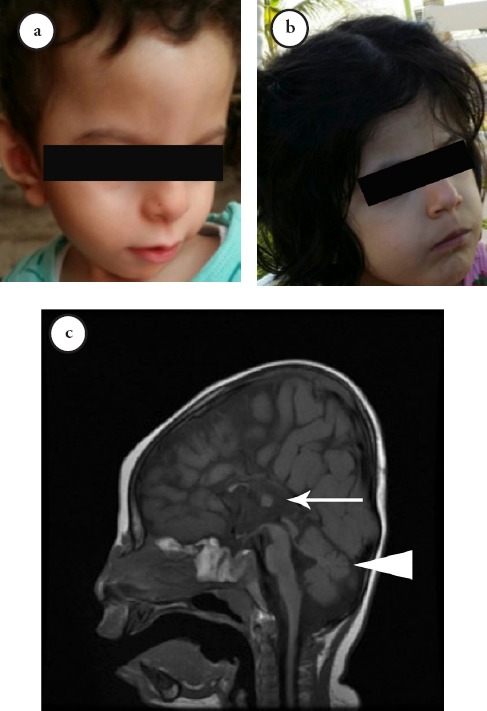
- Clinical features of the patients. **a, b**) The craniofacial features include dysmorphism in the form of wide forehead, hypotelorism, and upturned nose. **c**) The brain MRI indicates complete agenesis of the corpus callosum (white arrow) with inferior vermis hypoplasia (arrowhead).

The neuro imaging revealed multiple brain abnormalities. An MRI in patients III-6 and III-8 demonstrated complete agenesis of the corpus callosum, hypoplasia of the inferior vermis, and thin optic nerves, associated with mild delayed myelination (**Figure 2**), while in patient III-7 (**Figure 2**), it showed thick corpus callosum and inferior vermis hypoplasia.

## Discussion

In humans, the incidence of balanced chromosome translocations is approximately one in 500. Balanced reciprocal translocations are associated with a 50% risk of spontaneous abortions and a 20% risk of genetic abnormalities.^8^ Unbalanced translocations involving chromosome 1 and 7 are well-recognized cytogenetic abnormalities in hematological malignancies.^1^ So far, only 7 patients with the t(1;7)-related neurological phenotype are reported in the literature.^2^-^7^ In this paper, we report 3 more patients. Our patients’ phenotype is dominant by hypotonia, developmental delay, microcephaly, seizures, and particular neuroimaging findings.

Two neurological phenotypes associated with t(1;7) can be described. The first phenotype is associated with behavioral disorders in the form of early onset schizophrenia, and autistic behavior. Neuroimaging in these cases is reported as normal. This phenotype may be explained by possible susceptibility loci for specific language disorder, or autism, reported from several linkage studies, both of which are positioned around 1q31-q34.^3^,^4^,^7^ The second phenotype, as in our patients, is associated with brain malformation. Two types of malformations have been reported: holoprosencephaly, as described by Chuang et al^2^ and Schinzel^5^,^6^ and abnormal corpus callosum and cerebellum as in our patients. The occurrence of brain malformation in t(1;7) may be explained by the involvement of several genes. Deletion of 7q 36 clearly involved the sonic hedgehog gene; and haploinsufficiency of the sonic hedgehog gene has been shown to be the responsible molecular event in some cases of holoprosencephaly.^9^ The sonic hedgehog gene exerts a strong influence on the differentiation of ventral and medial structures of the prosencephalon, and the defective expression resulting from a mutation of this gene is thought to be the molecular basis of the human malformation holoprosencephaly with a broad spectrum of clinical severity. The defect in cleavage also influence the development of other cerebral structures that occur later in ontogenesis resulting in an abnormality in the formation of the corpus callosum, and various migrational disorders, such as dysplasia.

In conclusion, we expand the number of patients with t(1;7)-related neurological disease to 10, and confirm the core features of the neurological phenotype of the disease as early psychiatric features or developmental delay, microcephaly, and seizures associated with brain malformation. We suggest that neuroimaging in these patients reveals a characteristic pattern that could help with early diagnosis. Further research is needed to understand the signaling network, and to shed light upon the role of chromosomes 1 and 7 in the developing brain.

## References

[1] Sanada M, Uike N, Ohyashiki K, Ozawa K, Lili W, Hangaishi A (2007). Unbalanced translocation der(1;7)(q10;p10) defines a unique clinicopathological subgroup of myeloid neoplasms. Leukemia.

[2] Chuang L, Kuo PL, Yang HB, Chien CH, Chen PY, Chang CH (2003). Prenatal diagnosis of holoprosencephaly in two fetuses with der (7)t(1;7)(q32;q32) pat inherited from the father with double translocations. Prenat Diagn.

[3] Gordon CT, Krasnewich D, White B, Lenane M, Rapoport JL (1994). Translocation involving chromosome 1 and 7 in a boy with childhood-onset schizophrenia. J Autism Dev Disord.

[4] McPherson EW, Laneri G, Clemens MM, Kochmar SJ, Surti U (1997). Apparently balanced t(1;7)(q21.3;q34) in an infant with Coffin-Siris syndrome. Am J Med Genet.

[5] Schinzel A (1984). Cyclopia and cebocephaly in two newborn infants with unbalanced segregation of a familial translocation rcp (1;7)(q32;q34). Am J Med Genet.

[6] Schinzel A (1986). Letter to the editor:a further case of cyclopia due to unbalanced segregation of a previously reported rcp(1;7)(q32;q34) familial translocation. Am J Med Genet.

[7] Yan WL, Guan XY, Green ED, Nicolson R, Yap TK, Zhang J (2000). Childhood-onset schizophrenia/autistic disorder and t(1;7) reciprocal translocation:identification of a BAC contig spanning the translocation breakpoint at 7q21. Am J Med Genet.

[8] Hook EB, Hamerton JL, Hook EB, Porter IH (1977). The frequency of chromosome abnormalities detected in consecutive newborn studies –differences between studies –results by sex and by severity of phenotypic involvement. Population Cytogenetics Studies in Humans.

[9] Solomon BD, Bear KA, Wyllie A, Keaton AA, Dubourg C, David V (2012). Genotypic and phenotypic analysis of 396 individuals with mutations in Sonic Hedgehog. J Med Genet.

